# Microbial Contamination and Antimicrobial Resistance in Use of Ophthalmic Solutions at the Department of Ophthalmology, Jimma University Specialized Hospital, Southwest Ethiopia

**DOI:** 10.1155/2019/5372530

**Published:** 2019-04-15

**Authors:** Lemlem Tamrat, Yeshigeta Gelaw, Getenet Beyene, Addisu Gize

**Affiliations:** ^1^Department of Ophthalmology, Saint Paul's Hospital Millennium Medical College, Addis Ababa, Ethiopia; ^2^Department of Ophthalmology, College of Medicine and Health Sciences, Bahir Dar Universisty, Bahir Dar, Ethiopia; ^3^Department of Laboratory Science, Faculty of Health and Medical Sciences, Jimma University, Jimma, Ethiopia; ^4^Department of Microbiology, St. Paul's Hospital Millennium Medical College, Addis Ababa, Ethiopia

## Abstract

**Background:**

Eye drops are most frequently used medications in ophthalmology. The carriage of pathogenic organisms to eyes through the agency of eye drops has presented a serious problem for several decades. The objective of this study was to determine the magnitude of contamination and pattern of antimicrobial resistance of in-use ophthalmic solutions.

**Method:**

A cross-sectional study was conducted at the Department of Ophthalmology, Jimma University Specialized Hospital (JUSH), Southwest Ethiopia, from June to December 2015. Samples from all ophthalmic solutions from outpatient department, operation theaters, and wards after an average duration of use of two weeks were taken. Samples were cultured and organisms were identified; antimicrobial susceptibility testing was performed using standard microbial identification techniques. The data were analyzed using SPSS software. Chi-square test was done and associations were taken as significant if *P* < 0.05.

**Result:**

The rate of contamination of eye drops in the study setup was found to be 51/70 (72.8%). Frequency of contamination of eye drops found was to be statistically associated with the duration of use of eye drops. Contaminations of eye drops were high among patients who self-administer the medications and those individuals who apply the medication less frequently. Tips of the bottles were more often contaminated than the content of the eye drop. Majority of both Gram-positive and Gram-negative organisms were sensitive for most of the broad-spectrum antibiotics; however, there were a significant number of Gram-negative organisms resistant to almost all antibiotics used.

**Conclusion:**

There is high rate of contamination of eye drops in the setup (72.8%). Duration of use of eye drops is a significant factor associated with contamination. Knowing duration time of each container and patient education on eye drop administration technique are mandatory.

## 1. Background

Topical eye drop medications are the most frequently used medications in Ophthalmology. It can be prepared locally or can be bought from market. Commonly used eye drops include tetracaine, mydriatics, miotics, *β*-blockers, lubricants, and steroids [1, 2]. Eye drops are presumed sterile when first opened. Contaminated eye drops used for both diagnostic and therapeutic purposes are a potential cause of ocular infections. Bacterial ocular infections such as keratitis [2] and endophthalmitis can be caused by contaminated eye droppers [3]. The published contamination rate of in-use eye drops varies widely from 0.07% to 35.8% [2, 4].

Eye drop contamination increases with the duration of use [1, 5]. A dramatic increase in eye drop contamination appeared in many studies proportional to the use of frequencies [1, 6–8]. Vials containing an antimicrobial agent were less likely to be contaminated than vials without antimicrobials [9, 10]. Poor technique in administering the drops is another risk factor for contamination, especially if patients are self-administering their drugs [11, 12]. Elderly patients, with poor vision, coordination, and difficulty in fine grasping, which may lead to fingers touching the tip of the container or touching their eyes or skin with dropper, may again contaminate the container [5, 13]. Contact with fingers or lids, ciliaries, conjunctiva, or cornea is the possible cause of contamination even if instilled by healthcare professionals. The bottle tips are more often contaminated than the solution [5, 14].

Commonly identified Gram-positive bacteria in different reports include coagulase-negative *Staphylococci* [15], *Corynebacterium* species, *Propionibacterium* species, as well as *Staphylococcus aureus*, *Bacillus* species, *Micrococcus* species, and *Enterobacter* species [2, 16]. Potentially pathogenic Gram-negative organisms were significantly isolated from all medication sites than Gram-positive organisms. Commonly identified ones are *Serratia marcescens*, *Enterobacter aerogenes*, *Pseudomonas*, and *Proteus* [9, 17]. There are some studies noting high frequencies of fungal contamination in the caps of the eye drop products [2, 18]. Apart from the risk of infection, bacterial contamination of eye drops may alter the pH of the solution and therefore reduce the efficacy of the drug [10, 19]. Even though medication contamination is clinically important, little attention has been paid to the proper and aseptic method of preparation of eye drops in this specialized teaching hospital and limited studies exist in the country in general [15, 17]. Therefore, the aims of this study were to determine the magnitude and pattern of microbial contamination of in-use eye drops at the Department of Ophthalmology, Jimma University, Ethiopia.

## 2. Materials and Methods

The study was a cross-sectional hospital-based study which was undertaken from June to December 2015. All multidose eye drop vials in use by patients admitted in the outpatient department, operation theaters, and eye ward during the study period were taken for the study after an average use for two weeks. For the purpose of this study, commonly used eye drops were labeled with its first date of opening preparation. The number of days since the bottles were opened and the visual appearance of the bottles were noted on the data collecting format. A check list was used to record the sociodemographic information and the reason for the use of eye drops.

### 2.1. Sample Size and Sampling Technique

Sample size was calculated based on the single population proportion formula, considering the prevalence rate of 6% from the previous study conducted in Kenya [3]. Expected margin of error (*d*) was 0.05, and confidence interval (*z*) was 95%. A total of 70 eye drop vials were used consecutively consenting patients, who attended the Department of Ophthalmology, Jimma University, Ethiopia.

### 2.2. Specimen Collection, Transportation, and Microbial Isolation

All eye drop bottle samples were taken to the Department of Microbiology, Jimma University Specialized Hospital (JUSH), and were inoculated on the same day in order to minimize the effects of storage time and the clinical condition as far as possible. For the dropper tip, a sterile cotton swab moistened in sterile normal saline was used before wiping the nozzle tip of the eye drop containers and then inoculated onto blood agar plate (BAP), mannitol salt agar (MSA), MacConkey, chocolate agar, and Sabouraud Dextrose. For the residual eye drop, after cleaning the tip with 70% alcohol, the bottle was shaken well and one drop of the solution was directly inoculated by inverting the container on blood agar, mannitol salt agar, MacConkey, chocolate agar, and Sabouraud Dextrose Agar and then spread across the plates with loop. Blood agar was used for isolation of both Gram-positives and Gram-negatives. mannitol salt agar was used for isolation of Gram-positive bacteria particularly Staphylococci species and McConkey agar was used for Gram-negatives, and chocolate agar for the *Haemophilus* species. For isolation of the fungus, Sabouraud Dextrose Agar (SDA) plates was used.

The BAP, MSA, and McConkey agar were incubated at 37°C for 24 hours. The chocolate plates were incubated at 37°C in CO_2_. The SDA plates were incubated at 30°C for up to 7 days and evaluated for growth on days 1, 5, and 10. All media and antibiotics were purchased and used from Oxoid, UK.

### 2.3. Bacterial Identification

Bacteria isolates were identified based on Gram reaction, colony characterization, and biochemical test. Biochemical tests, namely, indole, citrate, oxidase, H_2_S production, KIA, lysine decarboxylase, lactose fermentation, urea hydrolysis, and gas production, were performed for identification for Gram-negative isolates. Catalase, coagulase test, and haemolysis pattern on blood agar was used for identification of Gram-positive bacteria. Fungal isolates were identified based on colony morphology on SDA and Gram stain [2], and germ tube test was done to isolate *Candida* spp.

### 2.4. Antimicrobial Susceptibility Testing (AST)

AST for the isolated colonies were done with the Kirby–Bauer disc diffusion method, a recommended method by National Committee for Clinical Laboratory Standards (CLSI) [20].

With an inoculating loop, colonies were transferred into a tube containing 4 to 5 ml of a suitable normal saline which was incubated at 35°C until it achieves the turbidity of the 0.5 McFarland standards (usually 2 to 6 hours). The dried surface of a Müeller-Hinton agar plate was inoculated by streaking the swab over the entire sterile agar surface. The predetermined battery of antimicrobial disc was dispensed onto the surface of the inoculated agar plate. Each antimicrobial disc was pressed down to ensure complete contact with the agar surface. The discs were placed individually no closer than 24 mm from center to center. The following antibiotic discs were used for antibiotic susceptibility testing. Tetracycline hydrochloride (TE, 30 *µ*g), penicillin G (P, 10 *µ*g), erythromycin (E, 15 *µ*g), vancomycin (VA, 30 *µ*g), oxacillin (OX, 5 *μ*g), gentamicin (GN, 10 *µ*g), chloramphenicol (C, 30 *µ*g), norfloxacin (NOR, 5 *µ*g), ciprofloxacin (CIP, 5 *µ*g), and cefuroxime (CXMP, 30 *µ*g). Vancomycin discs were used for only Gram-positives and gentamicin discs only for Gram-negatives.

The plates were inverted and placed in an incubator set to 35°C within 15 minutes after the discs were applied. After 18 to 24 hours of incubation, each plate was examined. The diameters of the zones of complete inhibition (as judged by the unaided eye) were measured to the nearest whole millimeter, using sliding calipers or a ruler, which is held on the back of the inverted Petri plate. The sizes of the zones of inhibition were interpreted by referring to Performance Standards for Antimicrobial Susceptibility Testing (NCCLS M100-S12), and the organisms were reported as susceptible, intermediate, or resistant to the agents that have been tested [20]. The quality of the culture media and Gram stain was checked using a standardized reference strain of *Escherichia coli* (ATCC 25922) and *Staphylococcus aureus* (ATCC 25923).

Ethical clearance was secured from the Research and Ethical Review Committee of the College of Health Science, Jimma University. Verbal consent was obtained from individual patients owning the eye drops.

## 3. Results

A total of 70 eye drop vials were analyzed, 25 (35.7%) of the bottles were from patients admitted in the ward during the study period, and 45 (64.3%) were from the outpatient department. Twenty percent 5/25 patients reported that they apply the eye drops by themselves. On the contrary, 20 (80.0%) reported that others apply the eye drop for them, and from these, 15 (75.0%) of them were using contaminated eye drops (Fisher's exact value = 1.0) (Table 1).

**Figure 1:**
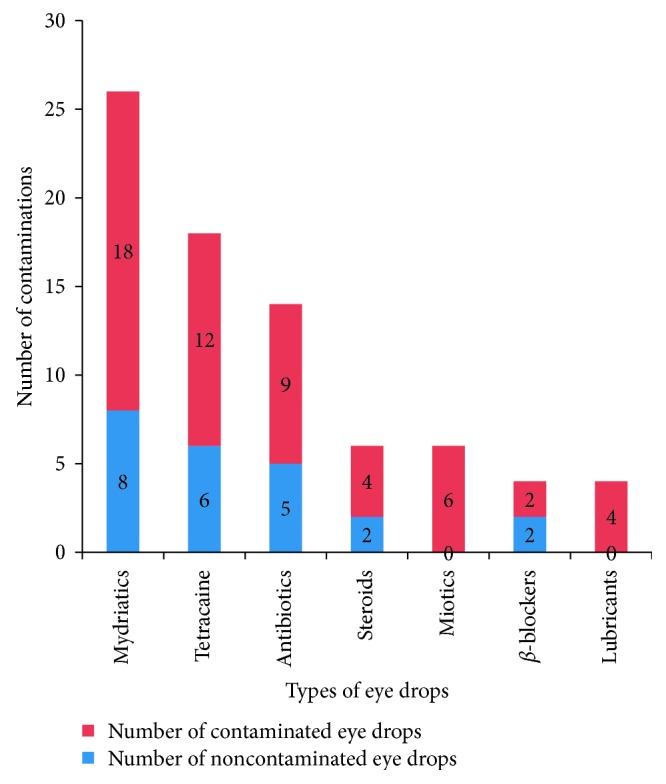
Types and number of contaminated commonly used eye drops contamination at Department of Ophthalmology, Jimma University from June to December 2015.

Most of the eye drops used in the department were mydriatics, tetracaine, and antibiotics 26 (37.1%), 18 (25.7%), and 14 (18.1%), respectively (Figure 1).

Contamination was found to be high in those vials without antibiotics but this was not statistically significant (*X*^2^ = 0.281, *P* value = 0.569). The pip of the eye bottle was more often contaminated than the drop (31 (60.8%) versus 2 (3.9%)).

Of the 70 eye drops, 51 (72.9%) were contaminated with one or more organisms. Most of the eye drops 58 (82.9%) were commercially bought. Forty-one (58.6%) of the eye drop vials were used by many patients, from these, 29 (70.7%) were contaminated. On the contrary, 29 (41.4%) of the eye drops were used by single patient and, from these, 22 (75.9%) of them were found to be contaminated. The majority of the eye drops, 41 (58.6%), were in use for 2–4 weeks, and 28 (40.0%) of them were in use for more than a month. Duration of use of eye drops and eye drop contamination were found to have a significant association (Chi-square = 4.462, *P* value = 0.035) (Table 2).

Of the 51 eye bottles contaminated, 43/51 (84.3%) of the bottles were bacteria, 28/51 (54.9%) of the bottles were fungus, and 20/51 (39.2%) were contaminated by both bacteria and fungus.

*Staphylococcus aureus* is the most common Gram-positive bacteria isolated followed by *Bacillus* species constituting 17 (60.7%) and 5 (17.9%), respectively. *Proteus* species and *Klebsiella* species were the most common Gram-negative isolates (Table 3).

The intermediate sensitive and resistant organisms were grouped under resistant category as it is listed in Table 4. All Gram-positive organisms found to be resistant to erythromycin and sensitive for ciprofloxacin. Most of the Gram-positive organisms were resistant to tetracycline (TTC) and penicillin and sensitive for chloramphenicol (CAF) and gentamicin. Most of the Gram-negative isolates were sensitive for gentamicin and chloramphenicol and resistant to vancomycin and oxacillin. It was also found out that one species of *Klebsiella* and one species of *Pseudomonas* were resistant for all the antibiotics tested (Table 4).

## 4. Discussion

In this study, contamination occurred in 51/70 (72.8%) of eye bottles used in the department. This is very high when compared to the results of other literature; for example, a study done in USA [5] and Kenya [3] reported 28% and 6% of contamination, respectively. This could be explained by poor handling, care, and follow-up given to eye drops used in the department. In line with other literatures [5, 17], the duration of use of eye drops was significantly associated with the contamination of eye drops (*P* < 0.05). The explanation could be the prolonged duration of use, which might increase the chance of contamination. From all kinds of eye drops analyzed, all miotics (pilocarpine) and lubricants were found to be contaminated. This is similar with a study done in Iran where pilocarpine is the highest rate of contamination than other eye drops [2]; this can be due to less common use of these eye drops, especially for communal use, which made them to be used for long time so that they will be exposed for contamination. It was also shown that contaminations of steroids were higher than that in another study [1]. Eye drop vials with antibiotics were found to be less frequently contaminated than vials without antibiotics. This might be due to the self-sterilizing effect of antibiotics. This finding is consistent with the other literature [9]. Unexpectedly, eye drops in use for a single patient were found to be more frequently contaminated than those in use for many patients. This could be due to negligence of patients to practice the instruction and the poor general hygienic condition of patients.

Self-administration was another issue related to contamination; when patients self-administer their drugs, they may inadvertently touch the eye lids with the tip and also with their own finger and cloths. The higher contamination rate on those eye drops in use less frequently (<4 times/day) contradicts the finding of one study done in the UK, which reported that eye drops used more frequently are at risk of contamination [9]. This might be related to the duration of use of these drugs, as drugs which are applied less frequently tend to be used for long time and thus they can be contaminated.

The most common Gram-positive organisms recovered were *Staphylococcus aureus* 17 (60.7%) and *Bacillus* species 5 (17.9%). This is comparable to other literatures [5]. A study done in the UK showed that *S. aureus* was the most common contaminant with percentage occurrence of 4.5% [9]. A study done in Kenya showed that 4/6 (66.7%) of the identified organisms were Gram-positive [3]. In this study, most of the Gram-positive organisms recovered were part of the normal flora of the conjunctiva. Normal resident flora of the conjunctiva and eye lids includes Gram-positive bacteria, including *S. aureus*, *Corynebacterium* species, *Propionibacterium* species, *Bacillus* species, *Micrococcus* species, and *Enterobacter* species [4, 5].

In this study, we found a high rate of contamination of ocular medications, particularly with potentially pathogenic Gram-negative organisms such as *Proteus* species, *Klebsiella* species, *Pseudomonas*, *Escherichia coli*, *Serratia* species, etc., that are not part of usual eye flora. *Proteus* spp. were present in 6/27 eye bottles, and *Klebsiella* species were seen in 4/27 vials in this study. Similar to many studies done in USA [1] we found Gram-negative organisms, specially *Proteus* species, *Serratia*, and *Pseudomonas* species, in a large number of eyedrops. This might be related to the poor technique and supervision of local eye drop preparation in the department and poor handling and use of eye drops by patients in the ward.

Majority of both Gram-positive and Gram-negative organisms were sensitive for most of the broad-spectrum antibiotics; however, there were a significant number of Gram-negative organisms found, which were resistant to almost all antibiotics used. This indicates that there exist pathogenic organisms nonresponsive to antibiotics in eye drop vials, which might contribute to ocular infection and also cross contamination of patients who share eye drop vials.

Fungus was isolated in 28 eye drop vials. This extent of contamination with fungus is not a small number for this setup; it is difficult to compare this to other studies because there are no literature studies on this issue.

## 5. Conclusions

There is high rate of contamination of eye drops in this setup. Gram-negative organisms more often contaminate the tip of eye drop rather than the drop. Most eye drop contaminants were sensitive for chloramphenicol, gentamicin, norfloxacin, and ciprofloxacin. Multiple drug resistance was seen among *Klebsiella* spp and *Pseudomonas* spp.

Marking the first date of opening on each container to know duration of time is mandatory so that it can be replaced on a regular basis. Patient education on eye drop administration technique, handling, and additionally safe technique in preparation of local eye drops needs to be considered by the responsible health professionals who work in this area.

## Figures and Tables

**Table 1:** Sociodemographic features and eye drop use versus contamination among patients admitted in the wards at Jimma University the Department of Ophthalmology from June to December 2015.

Sociodemographic characteristics		Contaminated *n*=19	Noncontaminated *n*=6	Total	*P* value
Age	<40 yrs	12 (75.0%)	4 (25.0%)	16 (64.0)	1.0^*∗*^
	≥40 yrs	7 (77.8%)	2 (22.2%)	9 (36.0%)	

Sex	Male	11 (73.3%)	4 (26.7%)	15 (60.0%)	1.00^*∗*^
	Female	8 (80.0%)	2 (20.0%)	10 (40.0%)	

Marriage	Married	11 (68.8%)	5 (31.2%)	16 (64.0%)	0.364^*∗*^
	Unmarried	8 (88.9%)	1 (11.1%)	9 (36.0%)	

Occupation	Employed	12 (75.0%)	4 (25.0%)	16 (64.0%)	1.00^*∗*^
	Unemployed	4 (44.4%)	5 (55.6%)	9 (36.0%)	

Education	Illiterate	13 (92.9%)	1 (7.1%)	14 (56.0%)	0.056^*∗*^
	Literate	6 (54.5%)	5 (45.5%)	11 (44.0%)	

Residence	Urban	15 (88.2%)	2 (11.8%)	17 (68.0%)	0.059^*∗*^
	Rural	4 (50.0%)	4 (50.0%)	8 (32.0%)	

Technique of drug administration	Self-administration	4 (80.0%)	1 (20.0%)	5 (20.0%)	1.0^*∗*^
	Non-self-administration	15 (75.0%)	5 (25.0%)	20 (80%)	

Reason of use of eye drops	Postoperative	7 (87.5%)	1 (12.5%)	8 (32.0%)	0.09
	Glaucoma	1 (50.0%)	1 (50.0%)	2 (8.0%)	
	Infection	10 (71.4%)	4 (28.6%)	14 (56.0%)	
	Lubrication	1 (100.0%)	0	1 (4.0%)	

Frequency of use of eye drops	<4 times/day	13 (81.2%)	3 (18.8%)	16 (64.0%)	0.63^*∗*^
	≥4 times/day	6 (66.7%)	3 (33.3%)	9 (36.0%)	

^*∗*^Fisher's exact test.

**Table 2:** Features of eye drops used in the Department of Ophthalmology, Jimma University, from June to December 2015.

Eye drop characteristics	Contaminated *n*=51	Noncontaminated *n*=19	Total	*X* ^2^	*P* value
*Place of use*					
Wards	18 (75.0%)	6 (25.0%)	24 (34.3%)	0.29	0.86
OPD	25 (73.5%)	9 (26.5%)	34 (48.6%)		
OR	8 (66.7%)	4 (33.3%)	12 (17.1%)		

*Expiry date*					
Not expired	23 (45.1%)	10 (52.6%)	33 (47.1%)	3.11	0.21
Expired	16 (31.4%)	8 (42.1%)	24 (34.3%)		
Not marked	12 (23.5%)	1 (5.3%)	13 (18.6%)		

*Duration of use*					
≤4 weeks	26 (63.4%)	15 (36.6%)	41 (58.6%)	4.46	0.04
>4 weeks	25 (86.2%)	4 (13.8%)	29 (41.4%)		

*Preparation of drops*					
Locally made	11 (91.7%)	1 (8.3%)	12 (17.1%)	2.59	0.11
Commercially bought	40 (69.0%)	18 (31.0%)	58 (82.9%)		

*Use of eye drops*					
Single patient use	22 (75.9%)	7 (24.1%)	29 (41.4%)	0.23	0.63
Many patient use	29 (70.7%)	12 (29.3%)	41 (58.6%)		

*Appearance*					
Clean	48 (71.6%)	19 (28.4%)	67 (95.7%)		
Dirty	3 (100.0%)	0	3 (5.3%)		

OPD = outpatient department; OR = operation room.

**Table 3:** Gram-positive and Gram-negative bacteria species cultured from the contaminated eye drop bottles at the Department of Ophthalmology, Jimma University from June–December 2015.

Organisms isolated		Number (%)
Gram-positive bacteria	*Staphylococcus aurous* (CPS)	17 (60.7%)
	*Bacillus* spp	5 (17.9%)
	*Staphylococcus saprophyticus* (CNS)	3 (10.7%)
	*Staphylococcus epidermidis*	2 (7.1%)
	*Corynebacterium* spp	1 (3.6%)
Total		28

Gram-negative bacteria	*Proteus* spp	5 (17.9%)
	*Klebsiella* spp	5 (17.9%)
	*Pseudomonas* spp	2 (7.1%)
	*Serratia* spp	2 (7.1%)
	*Morganella morganii*	2 (7.1%)
	*Citrobacter* spp	2 (7.1%)
	*E*. *coli*	2 (7.1%)
	*Edwardsella*	2 (7.1%)
	*Providencia*	2 (7.1%)
	*Salmonella* spp	2 (7.1%)
	*Acinetobacter* spp	2 (7.1%)
Total		28

**Table 4:** Resistance pattern of Gram-positive and Gram-negative isolates from eye drop bottles at the Department of Ophthalmology, Jimma University from June–December, 2015.

Organisms	No	Antimicrobial agent									
		Penicillin	Tetracycline	Chloramphenicol	Gentamicin	Oxacillin	Vancomycin	Norfloxacin	Erythromycin	Ciprofloxacin	Cefuroxime
*S. aureus*	17	17	17	4	5	7	12	4	17	0	12
*Bacillus* spp	5	5	5	1	1	4	3	1	5	0	5
*S. saprophyticus*	3	3	3	2	0	2	2	0	3	0	2
*S. epidermidis*	2	1	2	1	1	0	0	0	2	0	1
*Corynebacterium* spp	1	1	1	0	1	1	0	1	1	0	1
*Proteus* spp	5	5	5	0	0	5	5	0	5	0	3
*Klebsiella* spp	5	5	5	2	2	4	4	2	5	1	4
*Pseudomonas* spp	2	2	2	1	1	2	2	1	2	1	1
*Serratia* spp	2	2	2	0	0	2	2	0	2	0	1
*Morganella morganii*	2	1	2	1	0	2	1	1	2	0	1
*Citrobacter* spp	2	2	2	0	1	2	2	0	2	0	1
*E. coli*	2	2	2	1	0	2	2	1	2	0	2
*Edwardsiella* spp	2	2	2	1	0	2	2	0	2	0	2
*Providencia* spp	2	2	2	2	1	2	2	2	2	1	2
*Salmonella* spp	2	2	2	0	0	2	2	0	1	1	2
*Acinetobacter* spp	2	2	2	1	0	2	2	0	2	0	2

## Data Availability

The data used to support this study are available from the corresponding author upon request.

## Abbreviations

AST: Antimicrobial susceptibility testing
BAP: Blood agar plate
CAF: Chloramphenicol
CLSI: Clinical laboratory standards institutes
JUSH: Jimma University Specialized Hospital
MSA: Mannitol salt agar
SDA: Sabouraud Dextrose Agar plates
SPP: Species
TTC: Tetracycline.
